# Compressed Sensing in On-Grid MIMO Radar

**DOI:** 10.1155/2015/397878

**Published:** 2015-09-30

**Authors:** Michael F. Minner

**Affiliations:** Department of Mathematics, Drexel University, Philadelphia, PA 19104-0250, USA

## Abstract

The accurate detection of targets is a significant problem in multiple-input multiple-output (MIMO) radar. Recent advances of Compressive Sensing offer a means of efficiently accomplishing this task. The sparsity constraints needed to apply the techniques of Compressive Sensing to problems in radar systems have led to discretizations of the target scene in various domains, such as azimuth, time delay, and Doppler. Building upon recent work, we investigate the feasibility of on-grid Compressive Sensing-based MIMO radar via a threefold azimuth-delay-Doppler discretization for target detection and parameter estimation. We utilize a colocated random sensor array and transmit distinct linear chirps to a small scene with few, slowly moving targets. Relying upon standard far-field and narrowband assumptions, we analyze the efficacy of various recovery algorithms in determining the parameters of the scene through numerical simulations, with particular focus on the *ℓ*_1_-squared Nonnegative Regularization method.

## 1. Introduction

Multiple-input multiple-output (MIMO) radar systems have garnered significant interest in recent years for the purpose of accurately detecting targets. These systems incorporate multiple antennas to transmit signals to a target scene and receive and process the reflected echoes. Depending on the positioning of the antennas, that is, widely separated or colocated, they can provide enhanced target detection and parameter estimation. In particular, a colocated MIMO radar system which transmits waveforms with distinct frequencies can yield improved spatial resolution over similar setups, such as phased-array radar systems. See [1] for a detailed analysis of MIMO radar.

Also in recent years, the advancements of Compressive Sensing, as developed in [2–4], have attracted wide-spread attention as a means of efficiently recovering sparse (or compressible) signals. The essential problem of Compressive Sensing is to construct an approximation to a sparse vector **x** ∈ *ℂ* ^*N*^, where *N* is large, from a minimal number of linear measurements **y** = **A** **x**, where **A** ∈ *ℂ* ^*m*×*N*^ with *m* ≪ *N*, in a stable and robust manner. One would not expect such reconstruction to be possible due to the fact that *m* < *N*; however, the additional a priori knowledge that **x** is sparse allows for such recovery. See [5] for an extensive mathematical treatment of Compressive Sensing.

A key finding of Compressive Sensing states that a random measurement matrix **A** ∈ *ℂ* ^*m*×*N*^ with *m* ≥ *Cs*ln⁡(*N*/*s*), where *C* is a constant and *s* is the sparsity of the signal, will satisfy the* Restricted Isometry Property* (RIP) with high probability. This result is a computationally significant improvement over the hefty Nyquist-rate because the RIP guarantees that any *s*-sparse signal **x** ∈ *ℂ* ^*N*^ can be recovered exactly. The signal **x** can be recovered by solving the convex optimization problem called* basis pursuit* or *ℓ*_1_ *-minimization*:

(1) minimize z1subject  to Az=y.

The efficacy of Compressive Sensing hinges on the sparsity of the signal one seeks to recover. Fortunately, the discretization of a target scene containing only a few point scatterers in the desired domain, such as angle, time delay, and Doppler, leads to an advantageous sparsity constraint since only a few of the bins in the domain contain a target in comparison to the total number of bins. Thus, techniques from Compressive Sensing have the potential to reduce costs without degrading resolution in detecting the reflectors. A great deal of research has been carried out in investigating the applicability of Compressive Sensing to a wide variety of radar systems and various aspects of radar signal processing. See [6, 7] for surveys of this field. Early work in this area includes [8, 9], while [10, 11] focus on MIMO radar in particular. We highlight two recent publications which serve as the basis for this work.

Strohmer and Wang [12] provide an excellent mathematical framework which incorporates Compressive Sensing for the recovery of on-grid targets in azimuth-range-Doppler via a MIMO radar system. They employ random sensor arrays and special waveforms, namely, the so-called Kerdock waveforms, in their setup and present a detailed mathematical analysis on the accurate detection of targets in such a setting. The target vector representing the scene is recovered via the* Debiased LASSO*, which is a variation of the well-known* LASSO*, from a number of measurements corresponding to the product of the number of receivers and the number of samples taken.

He et al. [13] devise an adaptive procedure to detect off-grid targets in azimuth and range with a MIMO radar system which features a uniform linear array (ULA) and transmits linear chirps with distinct frequencies. They highlight the performance of their algorithm with numerical simulations and compare the results to alternative recovery methods. These authors rely upon the orthogonality of their waveforms to obtain a number of measurements equal to the product of the number of transmitters, the number of receivers, and the number of samples taken.

This paper incorporates elements from both of these works. Namely, we utilize a random array MIMO radar system which transmits linear chirps and obtains a number of measurements equal to the product of the number of transmitters, the number of receivers, and the number of time samples in order to detect on-grid targets in the azimuth, time-delay, and Doppler domain. Various numerical simulations are performed in this framework for a small target scene containing only a few slowly moving point scatterers. We initially utilize several different algorithms for recovery and then focus solely on the *ℓ*_1_-squared Nonnegative Regularization (L1SQNN) from [14] due to its superior performance in comparison to the other selected methods. The choice of the random sensor array in place of a ULA is justified by a set of results directly comparing the two setups. We further analyze how changes in the sparsity level, signal-to-noise ratio (SNR), problem size, regularization parameters, and bandwidth can impact the reconstruction. A collection of MATLAB files, figures, and data sets from these simulations are available for download in the accompanying supplementary materials.

## 2. Problem Formulation

Consider a colocated MIMO radar system with *M* randomly positioned transmit antennas and *N* randomly positioned receiver antennas in the sensor array. Each of the transmitters repeatedly sends a waveform *s*_*m*_(*t*), for *m* = 1,…, *M*, which are orthogonal to each other and narrowband, to reflect off *P* point targets in a far-field scene and return to the *N* receiver antennas. These returning signals are observed over a duration *T*_*d*_. We discretize the scene in azimuth, delay (radial range), and Doppler (radial velocity) with *U* angle bins, *V* delay bins, and *W* Doppler bins and associated discretization steps Δ_*θ*_, Δ_*τ*_, and Δ_*υ*_. Hence, targets located exactly on the grid correspond to a location (*θ*_*k*_, *τ*_*k*_, *υ*_*k*_) = (*θ*_ref_ + *u*_(*k*)_Δ_*θ*_, *τ*_ref_ + *v*_(*k*)_Δ_*τ*_, *υ*_ref_ + *w*_(*k*)_Δ_*υ*_), where *k* = 1,…, *K*, with *K* = *UVW*, *θ*_ref_, *τ*_ref_, and *υ*_ref_ are reference values in the respective domains and where (*u*_(*k*)_, *v*_(*k*)_, *w*_(*k*)_) represents the *k*th bin in the discretization of the scene. These point targets possess nonzero complex reflectivity coefficients *x*_*k*_ and are assumed to be (slow) moving with constant velocities. If there is no target at grid point (*θ*_*k*_, *τ*_*k*_, *υ*_*k*_), then the associated *x*_*k*_ = 0. We let *S* ⊂ {1,2,…, *K*} denote the locations of the targets; that is, *S*≔{*k* ∈ [*K*] : *x*_*k*_ ≠ 0} and |*S*| = *P*.

We introduce the array manifolds for the small target scene:

(2) aθk=1,ei2π/λdt2θk,…,ei2π/λdtMθkT,bθk=1,ei2π/λdr2θk,…,ei2π/λdrNθkT,

where *λ* is the reference carrier wavelength, *dt*_*m*_, for *m* = 2,…, *M*, is the distance from the *m*th transmitter to the first transmitter, and *dr*_*n*_, for *n* = 2,…, *N*, is the distance from the *n*th receiver to the first receiver (we are using the approximation sin⁡(*θ*_*k*_) ≈ *θ*_*k*_ since we will only consider small angles, i.e., |*θ*_*k*_ | <0.1 radians). Hence, under the narrowband assumption and after orthogonal separation, the signal received from the *m*th transmitter at the *n*th receiver at time *t* is given by

(3) zmnt=∑k∈Sxkbnθkamθksmt−τkexp⁡−i2πυkt+emnt,

where *b*_*n*_(*θ*_*k*_) is the *n*th entry of **b**(*θ*_*k*_) and *a*_*m*_(*θ*_*k*_) is the *m*th entry of **a**(*θ*_*k*_) and *e*_*mn*_(*t*) is noise.

We employ Linear Frequency Modulated (LFM) chirps of the following form:

(4) $$\begin{array}{c} s_{m}\left(\;t\;\right)=\exp\left[\;i2\pi \left(\;\frac{\alpha }{2}t^{2}+f_{m}t\;\right)\;\right]I_{T}\left(\;t\;\right), \end{array}$$

where *α* is the chirp rate, *T* is the pulse duration, *f*_*m*_ = *f*_ref_ + *mαT* is the carrier frequency for a specified reference carrier frequency *f*_ref_, and *I*_*T*_(*t*) is the characteristic function on [0, *T*]. We suppose that the reference range for the scene is *R*_ref_; thus, the reference time delay is *τ*_ref_ = 2*R*_ref_/*c* and for LFM chirps we obtain the following:

(5) smt−τksm∗t−τref=ITt−τrefITt−τk·exp⁡−i2πfm+αt′τk−τref·exp⁡iπατk−τref2,

where *t*′ = *t* − *τ*_ref_ and *t*′ ∈ [0, *T*_*d*_]. The quadratic term is known as the residual video phase and can be removed according to [15].

Thus, after dechirping, the measurements we obtain between the *m*th transmitter and the *n*th receiver at time *t*′ take the following form:

(6) ymnt′∑k=1Kxkbnθkamθk·exp⁡−i2πfm+αt′τk−τref·exp⁡−i2πυkt′+τref+emnt.

Note that contributions to the summation only result from the *P* point targets; however, since it is unknown a priori which *x*_*k*_ are nonzero, we sum over all *K* possible locations. We consider the measurements at times *t*_*q*_′, for *q* = 1,…, *Q*. After substituting the appropriate expressions into (6), we have

(7) ymntq′∑k=1Kxkexp⁡i2πλdtm+drnθk·exp−i2πfm+αtq′τk−τref·exp⁡−i2πυktq′+τref+emntq′.

Our objective now is to recover {*x*_*k*_, *θ*_*k*_, *τ*_*k*_, *υ*_*k*_}_*k*=1_^*K*^ from the set of {*y*_*mn*_(*t*_*q*_′)}, where *m* = 1,…, *M*, *n* = 1,…, *N*, and *q* = 1,…, *Q*. Hence, we define, via the vectorization operation vec⁡(·), **y**≔vec⁡(*y*_*mn*_(*t*_*q*_′)), **e**≔vec⁡(*e*_*mn*_(*t*_*q*_)), and

(8) Ak≔vec⁡exp⁡i2πdtm+drnλθk−υktq′+τref·exp⁡−i2πfm+αtq′τk−τref,

which are all vectors of size *MNQ* × 1. We store **A**_*k*_'s via

(9) $$\begin{array}{c} A=\left(\begin{array}{cccc} A_{1} & A_{2} & \cdots & A_{K} \end{array}\right). \end{array}$$

Letting **x** = [*x*_1_, *x*_2_,…,*x*_*K*_]^*T*^, we arrive at the standard Compressive Sensing framework:

(10) $$\begin{array}{c} y=Ax+e. \end{array}$$

Here, **y** is the set of measurements we obtain from our measurement matrix **A**, the *P*-sparse target scene **x**, and the noise vector **e**. Thus, our goal is to recover **x** from **y** and **A** and in turn estimate *θ*_*k*_, *τ*_*k*_, and *υ*_*k*_ associated with each nonzero *x*_*k*_.

## 3. Numerical Simulations

Simulations were performed in MATLAB to investigate the efficacy of various reconstruction algorithms in recovering sparse vectors from (10). The following parameters remain unchanged throughout the simulations: the reference carrier frequency is *f*_ref_ = 10 GHz, the bandwidth of each transmitted signal is *B* = 15 MHz, the pulse duration is *T* = 2 *μ*s (hence, the chirp rate is *α* = 7.5 × 10^−12^ Hz/s), the azimuth angles range (in radians) from −0.1 to 0.1, the radial range values are between 990 m and 1010 m, and the target velocities range from 0 m/s to 30 m/s. We consider *M* = 5 transmitters, *N* = 5 receivers, *Q* = 20 time samples, *U* = 10 azimuth bins, *V* = 10 time-delay bins, and *W* = 10 Doppler bins (thus *K* = 1000 bins all together) for the majority of the simulations, but we also double all of these values to explore how increasing the problem size impacts the recovery. We also investigate how the bandwidth affects the reconstruction in the final experiment. For each simulation set, the sparsity level of the target varies over some fixed collection of values, a number of measurement matrices are generated for each sparsity level, and a specified number of random target vectors are generated for each matrix.

The transmit and receiver antenna positions are generated independently according to the uniform distribution on [0, *MN*/2] as in [12], while the locations of the point targets are chosen iteratively. A location in the azimuth-delay grid is selected at random; if the bin does not already contain a target, then a Doppler value is selected at random and the new azimuth-delay-Doppler location is added to the support of the target scene. Otherwise, a new azimuth-delay location is chosen at random and the process repeats until the target vector contains the correct number of point scatterers. The targets are each given a unit reflectivity coefficient. After the measurements are taken, they are corrupted by complex, circularly symmetric Gaussian noise, for a designated SNR level. Recovery is then performed with Orthogonal Matching Pursuit (OMP), Adaptive Inverse Scale Space (AISS) [16], *ℓ*_1_-squared Nonnegative Regularization (L1SQNN) [14], and/or *ℓ*_1_/*ℓ*_2_ Constrained Nonnegative Regularization (L1L2CNN) from the YALL1 software package [17] (in the case of no noise,* basis pursuit* with a nonnegativity constraint is selected from YALL1 in place of L1L2CNN). The Orthogonal Matching Pursuit is a well-known greedy method which seeks to reconstruct the vector by iteratively building its support, one entry at a time, and finding the vector on the same support which best fits the measurements at each step. The other methods are variants on* basis pursuit denoising* [18]:

(11) $$\begin{array}{c} \underset{z\in R^{N}}{\mathrm{minimize}}{\left\|\;z\;\right\|}_{1}+\nu {\left\|\;Az-y\;\right\|}_{2}^{2}. \end{array}$$

Specifically, the L1SQNN method solves

(12) minimizez∈RN z12+β2Az−y22subject  to z≥0,

while the L1L2CNN method solves

(13) minimizez∈RN z1subject  to Az−y2≤δ, z≥0,

where ‖**e**‖ ≤ *δ*. As the name implies, the AISS approach relies on Inverse Scale Space methods and Bregman Iterations to iteratively solve lower dimension problems in seeking a solution to (1). Since we have assumed the point scatterers possess a unit reflectivity, we utilize the nonnegativity constraints in L1SQNN and L1L2CNN; however, for scatterers with complex reflectivity coefficients, variants of these two methods can be employed which drop the nonnegative constraint. While this collection is by no means exhaustive, we selected these algorithms for the following reasons: OMP is commonly used and easy to implement, the YALL1 package is readily available online, and L1SQNN and AISS are both more recently developed methods.

A set of threshold levels is used to zero out the entries of the recovered vector which fall below the specified threshold in magnitude so that all nonzero entries after thresholding are classified as targets in the scene. Throughout the simulations, the following quantities are calculated and averaged for each sparsity level: the probability of detection, the probability of false alarm, the relative error, and the number of iterations and amount of time required for the algorithms to terminate. The probability of detection is calculated by dividing the number of correctly identified targets, after thresholding, by the number of true targets present in the scene. Similarly, the probability of false alarm is calculated by dividing the number of falsely identified targets, after thresholding, by the number of vacant locations in the scene. The relative error for a recovered vector postthresholding, denoted $\tilde{x}$, is simply ${\left\|\tilde{x}-x\right\|}_{2}/{\left\|x\right\|}_{2}$. For several simulation sets, we plot Receiver Operating Characteristic (ROC) curves [19], that is, the probability of detection plotted against the probability of false alarm, which illustrate how lowering the threshold level increases both the probability of detection and probability of false alarm.

## 4. Results

**Figure 1:**
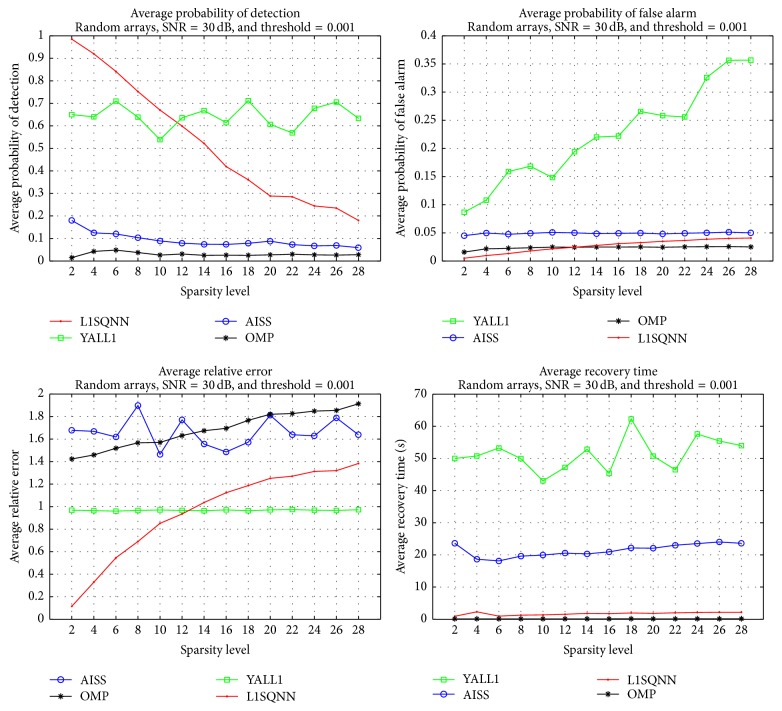
Comparison of OMP, AISS, L1SQNN, and L1L2CNN algorithms with fixed SNR = 30 dB and a threshold of 0.001.

The initial set of simulations were designed to provide a rough comparison of the performance of the previously discussed recovery algorithms. The SNR is fixed at 30 dB, a low threshold level of 0.001 is selected, and *β* = 30 is chosen as the parameter for L1SQNN, while the noise level is used in the L1L2CNN method from YALL1. A total of 100 simulations, that is, 10 matrices applied to 10 random target vectors, are performed for each sparsity level, which ranged from 2 to 28 with increments of 2. As shown in Figure 1, the L1SQNN algorithm offers superior recovery in comparison to the other three methods in all categories. Although the L1L2CNN algorithm provides a better probability of detection and lower relative error for higher sparsity counts, it comes at the cost of a higher probability of false alarm, by an order of magnitude, and much longer run times on average. Also, despite being not shown here, separate simulations reveal that the AISS method outperforms the L1SQNN method in the case of no noise, though it also requires a longer run time. Thus, due to the enhanced performance, we focus on the L1SQNN algorithm for the remaining simulations.

**Figure 2:**
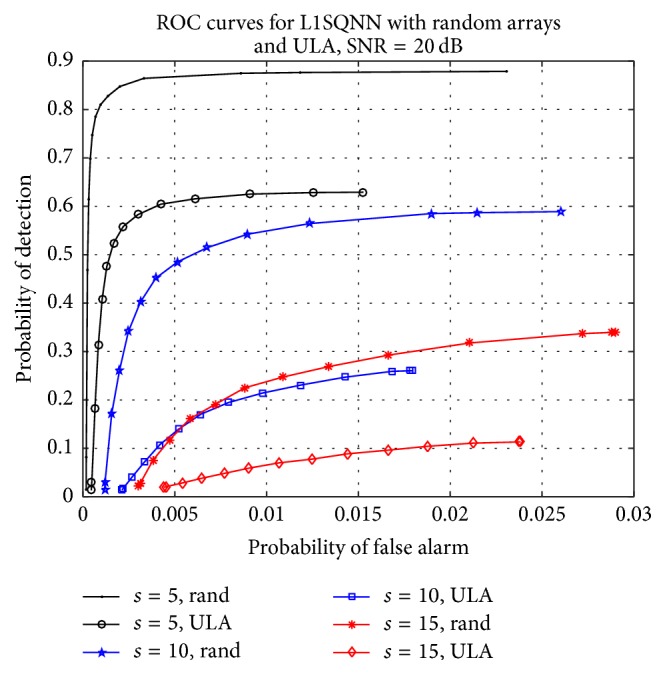
Comparison of ROC curves for L1SQNN with random arrays and ULA, SNR = 20 dB.

Next we consider both the random array and uniform linear array MIMO radar setups to justify the use of the random sensor arrays. The ULA system has receiver antennas positioned in a line with a uniform separation distance of *λ*_ref_, that is, the reference carrier wavelength *λ*_ref_ = *c*/*f*_ref_, and transmit antennas similarly positioned but with a uniform separation distance of 2*Nλ*_ref_. Here, the SNR is fixed at 20 dB, *β* = 9 is chosen as the parameter for L1SQNN, and a set of threshold values is taken from [0.0001,0.999]. The ULA-based matrix, which never changes since it is completely deterministic, and a random array-based matrix are each used to separately measure and recover the same vector at each step of the simulations. A total of 500 simulations, that is, 20 random array matrices (and 1 ULA matrix) applied to 25 target vectors, are performed for each sparsity level, which ranges from 5 to 15. As highlighted by the ROC curves in Figure 2, the random sensor array systems provide superior performance over the ULA system.

**Figure 3:**
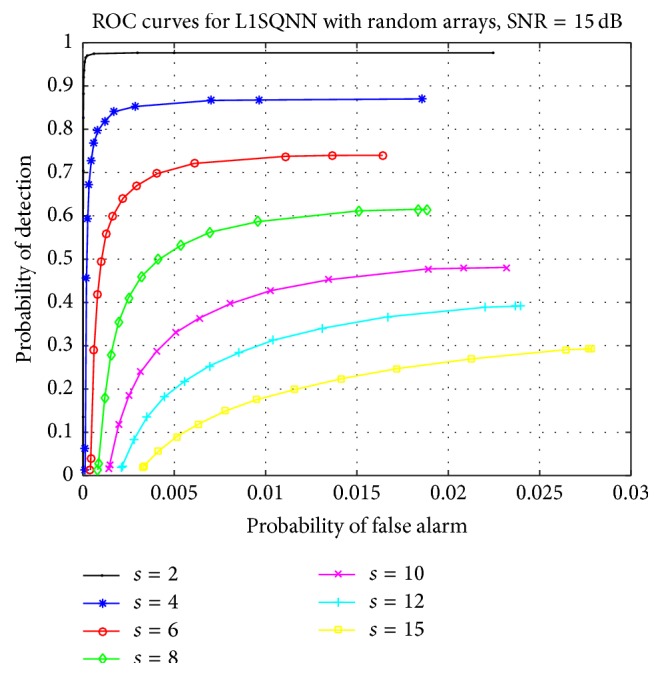
ROC curves for L1SQNN with random arrays, SNR = 15 dB, and various sparsity levels.

**Figure 4:**
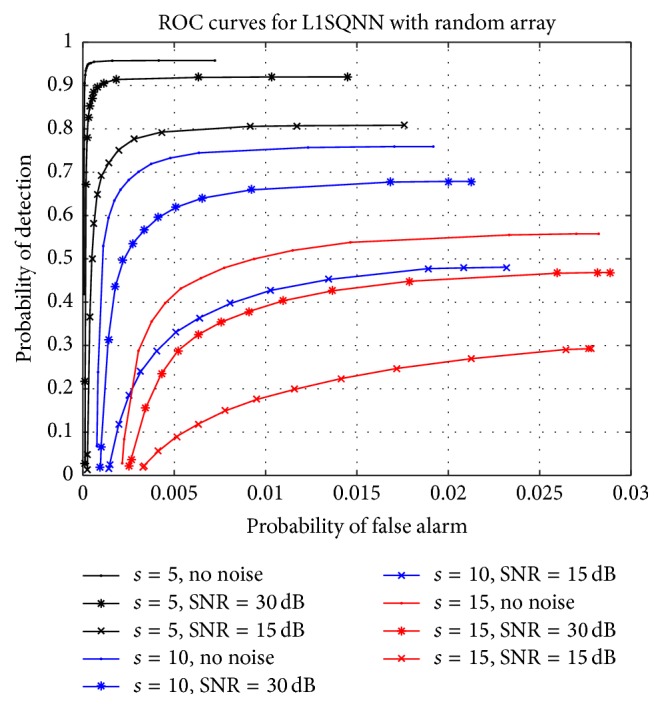
Families of ROC curves for L1SQNN with random arrays. Each family of curves corresponds to a different sparsity level (denoted by color), while individual curves within each family correspond to a different SNR (denoted by symbol).

Focusing on random sensor arrays, we examine how L1SQNN performs as the sparsity level increases. The SNR is decreased to 15 dB but remains fixed throughout the experiment, while *β* = 5 is chosen as regularization parameter. We perform 1000 simulations, that is, 25 matrices applied to 40 vectors, at each sparsity level, which now ranges from 2 to 15. The ROC curves in Figure 3 demonstrate a graceful decay in performance as the number of targets increases. Additionally, comparing the random array ROC curves from Figure 2, where the SNR was 20 dB, to the curves in Figure 3 with the same respective sparsity counts also illustrates a reasonable decline in performance as the SNR decreases. This decline is clearly highlighted in Figure 4 which presents the performance of L1SQNN at three different levels of SNR for three distinct sparsity levels.

**Figure 5:**
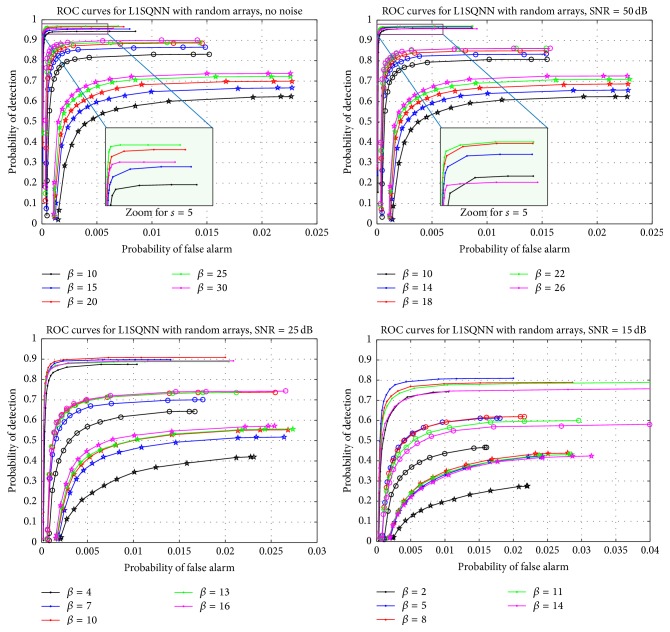
ROC curves for L1SQNN with random arrays at various levels of SNR and different values of the regularization parameter. The upper set of curves in each graph corresponds to a sparsity of 5 (dots), the middle set corresponds to a sparsity of 8 (circles), and the lower set corresponds to a sparsity of 12 (stars). The figures for the noiseless case and for SNR = 50 dB each feature a magnified view of a portion of the ROC curves when the sparsity is 5.

**Figure 6:**
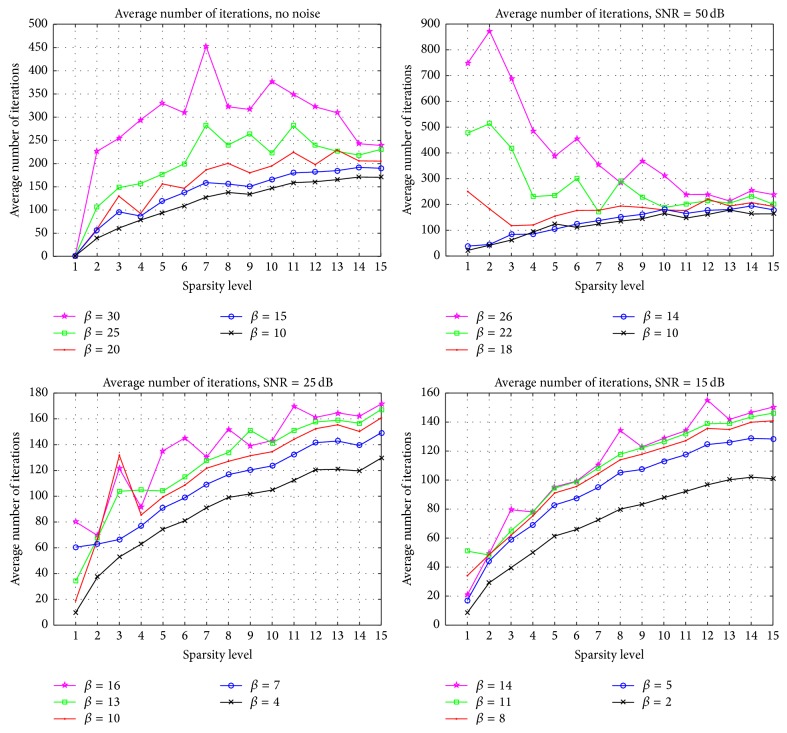
The average number of iterations versus the sparsity at distinct SNR levels and with different values of the regularization parameter for L1SQNN with random arrays.

For the next set of simulations, we investigate how changing the parameter of L1SQNN impacts its performance with random sensor arrays. As before, we run 400 simulations for each sparsity level with a set of threshold values taken from [0.0001,0.999]; however, we consider different levels of SNR separately. For each SNR, we perform the recovery with distinct values of the regularization parameter *β*. The results are displayed in Figures 5 and 6. Each subplot in Figure 5 contains separate families of ROC curves which correspond to the sparsity levels of 5 (dots), 8 (circles), and 12 (stars), respectively. Since the curves within each family represent a different value of the parameter *β*, these figures indicate that, for a fixed SNR, the value of *β* should increase to enhance performance as the sparsity increases. Furthermore, one can observe from Figure 5 that as the SNR rises, the regularization parameter should increase to improve performance overall. Comparing the plots in Figure 6 reveals, for select parameter values, a sharp increase in the average number of iterations needed for the method to terminate while moving from the noise-free scenario to the case where a small amount of noise corrupts the measurements, particularly at low sparsity levels. As the SNR continues to deteriorate, these apparent differences diminish. Figures 5 and 6 highlight the importance of fine-tuning the regularization parameter to attain the desired probability of detection or probability of false alarm. However, if the SNR and sparsity level are a priori unknown, then more advanced techniques may be used to estimate these quantities and update the parameter accordingly.

**Figure 7:**
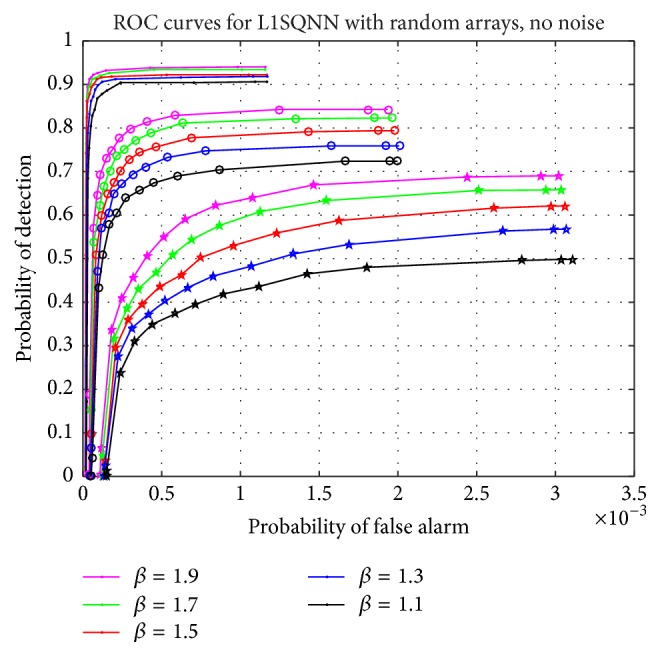
Comparison of various parameter values, *β*, in L1SQNN for the larger random array measurement matrices of size 4000 × 8000 with no noise. The upper set of curves corresponds to a sparsity of 5 (dots), the middle set corresponds to a sparsity of 8 (circles), and the lower set corresponds to a sparsity of 12 (stars).

The previous simulations are repeated on a smaller scale but for a larger problem: the numbers of transmitters, receivers, sample times, and bins in each domain are doubled; hence, the dimensions of the measurement matrix have increased from 500 × 1000 to 4000 × 8000. However, only 100 simulations are performed; that is, 10 random sensor array measurement matrices are applied to 10 random target vectors. The results for the noise-free scenario are presented in Figure 7. The most noticeable difference between the ROC curves of Figure 7 and those in the noise-free plot from Figure 5 is the order of magnitude decrease in the probability of false alarm for the larger problem. This is appropriate given the increase in the number of bins from 1000 to 8000. Comparing these figures also exposes a heightened sensitivity to the regularization parameter since smaller variations in *β* lead to more pronounced changes in the ROC curves, as displayed in Figure 7. Despite being not shown here, the number of iterations is consistent with previous simulations, but the average amount of time needed for the algorithm to terminate is significantly longer, by at least an order of magnitude, depending on the sparsity level, and increases for greater values of *β*.

**Figure 8:**
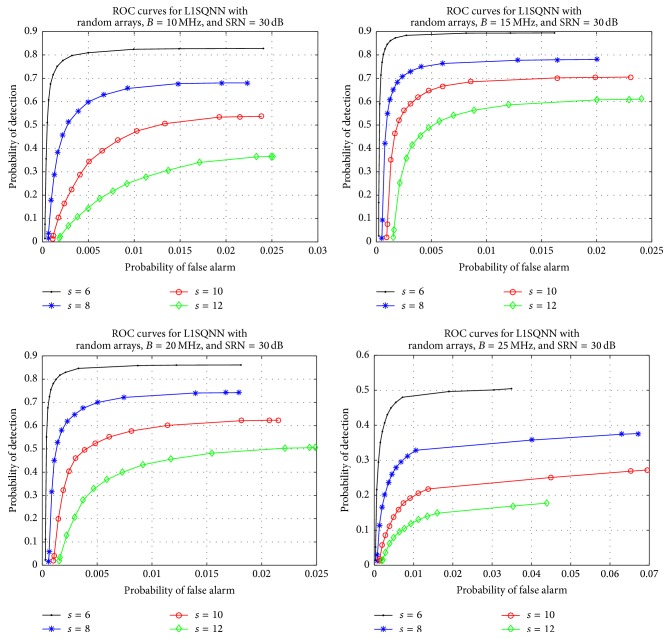
Comparison of various bandwidth values, *B*, in L1SQNN with random arrays, SNR = 30 dB, and various sparsity levels.

As a final experiment, we explore how changing the bandwidth impacts the performance. We select a set of values for the bandwidth, *B*, and at each value run 800 simulations for a predetermined collection of sparsity levels. The reconstruction is performed with a fixed regularization parameter, *β* = 15, along with a set of threshold values taken from [0.0001,0.999] and a constant SNR of 30 dB. The results are displayed in Figure 8. The subplots in Figure 8 correspond to the bandwidth values of 10, 15, 20, and 25 MHz, respectively, while the curves within each subplot represent a different sparsity level. Noting how each ROC curve for a fixed sparsity changes across the subplots, one can observe that as the bandwidth rises, the performance improves and then decays. Further simulations indicate that the performance improves again briefly as the bandwidth continues to increase but then drops off sharply.

The results from each of these sets of simulations indicate the following: for the specified physical parameters, this sparse vector recovery problem requires a significantly low sparsity level to achieve meaningful performance. As the sparsity level increases from just a few scatterers and as the SNR decreases from the noiseless setting, the performance consistently decays, despite being typically in a graceful manner. Although increasing the number of bins in each domain does not greatly inhibit the results, it does lead to a significant increase in the run time. This is due to the high computational complexity which results from discretizing in azimuth, delay, and Doppler instead of simply one or two of these domains.

## 5. Conclusion

We have combined elements from recent work in [12, 13] to further investigate the applicability of Compressive Sensing to a MIMO radar system. Specifically, we have considered a random array MIMO radar system which transmits linear chirps and utilizes orthogonal separation and dechirping to acquire additional measurements while probing a small target scene in the azimuth-delay-Doppler domain. The various simulations, which are available for download, demonstrate superior performance when only a few point scatterers are present in the scene. The *ℓ*_1_-squared Nonnegative Regularization method from [14] provides enhanced recovery in the presence of noise over the other algorithms considered; however, as is always the case, the regularization parameter must be finely tuned to achieve a desired false alarm rate. This work is intended as an initial step in exploring the feasibility of applying techniques from Compressive Sensing to the off-grid MIMO radar problem in the azimuth, time-delay, and Doppler domain.

## Supplementary Material

The Supplementary Material accompanying this paper is a TAR file containing the MATLAB files needed to reproduce the results of the paper. These include the exact code used in each experiment, the resulting data sets from each experiment, and the code to generate the plots for each of these data sets.
